# Correction: Dissecting genetic and sex-specific sources of host heterogeneity in pathogen shedding and spread

**DOI:** 10.1371/journal.ppat.1009415

**Published:** 2021-03-08

**Authors:** PLOS Pathogens staff

S1 Data is omitted from the list of Supporting Information. It can be viewed below. The full legend for this file is:

## Supporting information

S1 Data Data and R code for all statistical analyses and to generate all figures. (ZIP)
