# Supplementary figures and images for: Dissecting genetic and sex-specific sources of host heterogeneity in pathogen shedding and spread
Source: PLoS Pathog. 2021 Jan 19;17(1):e1009196. doi: 10.1371/journal.ppat.1009196 (PMC7846003; doi:10.1371/journal.ppat.1009196)

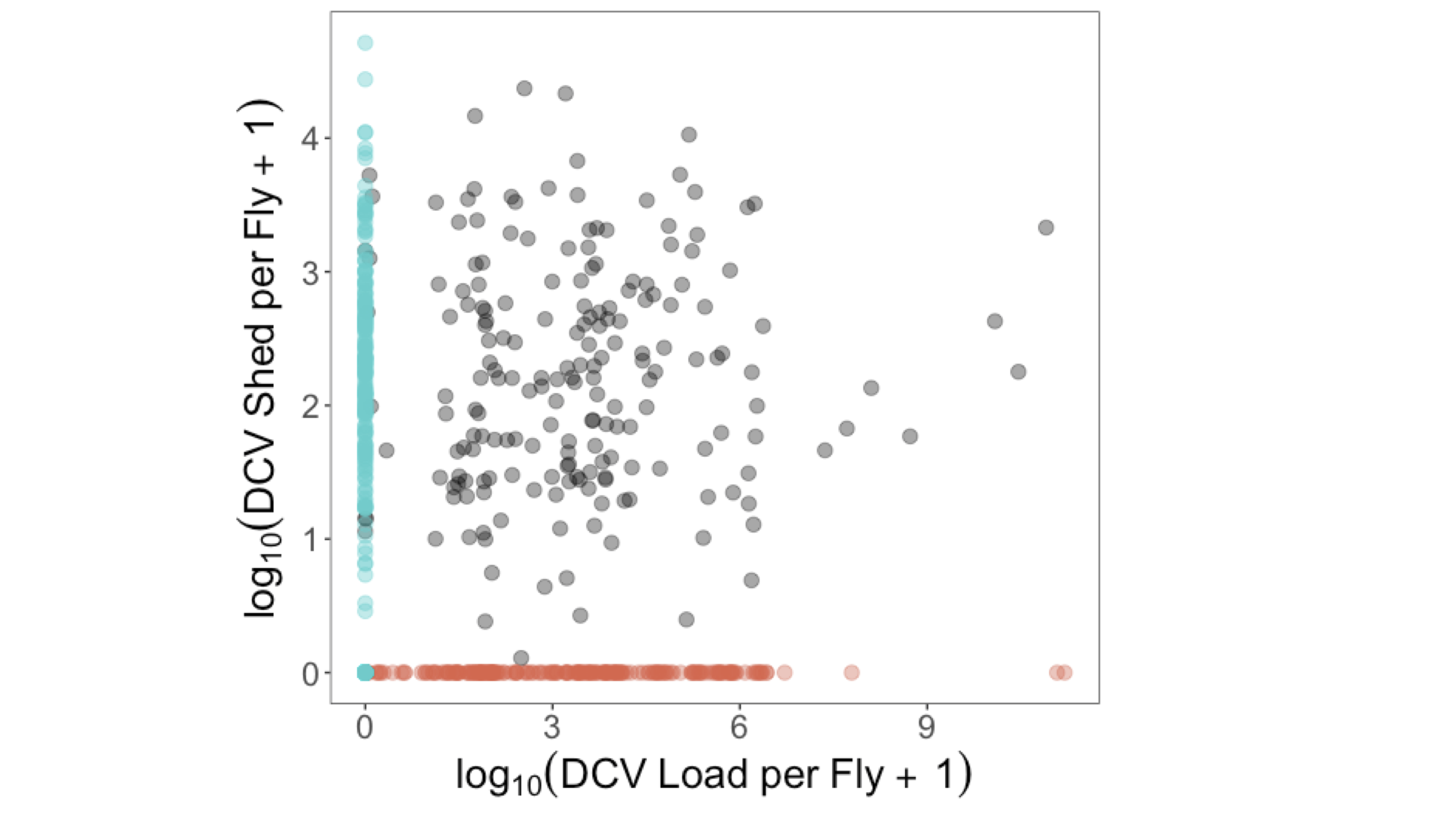

Supplement: S1 Fig — The two distinct phenotypes, where individuals show a zero-value for shedding or load and a positive-value for the other trait, are marked by red (supersponges) or blue (supershedders). (TIF) [file ppat.1009196.s001.tif]

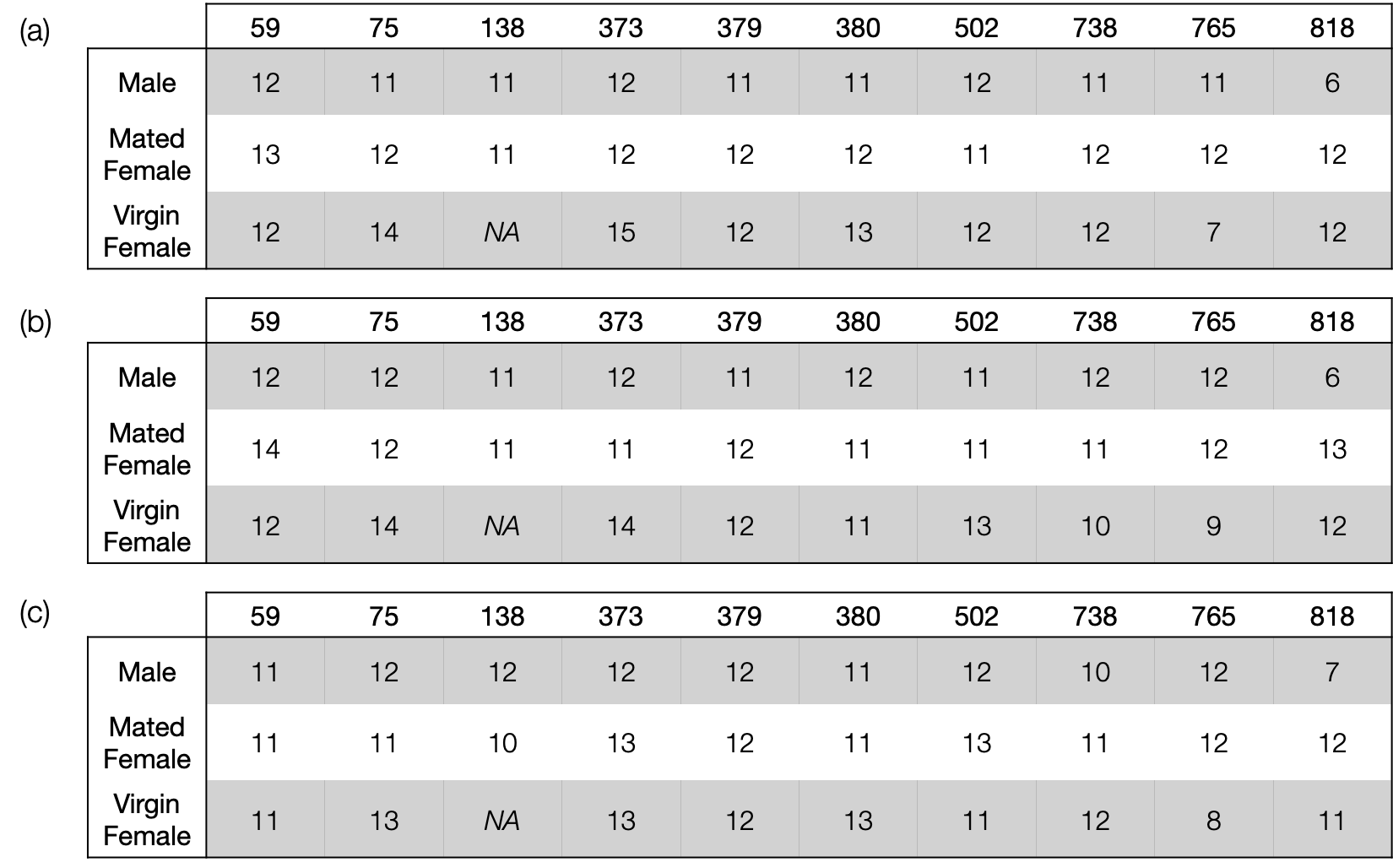


**S2 Table** – The number of viral load samples for each treatment group (a) 1 DPI, (b) 2 DPI and (c) 3 DPI.

Supplement: S2 Table. The number of viral load samples for each treatment group (a) 1 DPI, (b) 2 DPI and (c) 3 DPI — (DOCX) [file ppat.1009196.s003.docx]
