# Supplementary material for: Dissecting genetic and sex-specific sources of host heterogeneity in pathogen shedding and spread
Source: PLoS Pathog. 2021 Jan 19;17(1):e1009196. doi: 10.1371/journal.ppat.1009196 (PMC7846003; doi:10.1371/journal.ppat.1009196)
Supplement: S1 Table — (DOCX) [file ppat.1009196.s002.docx]

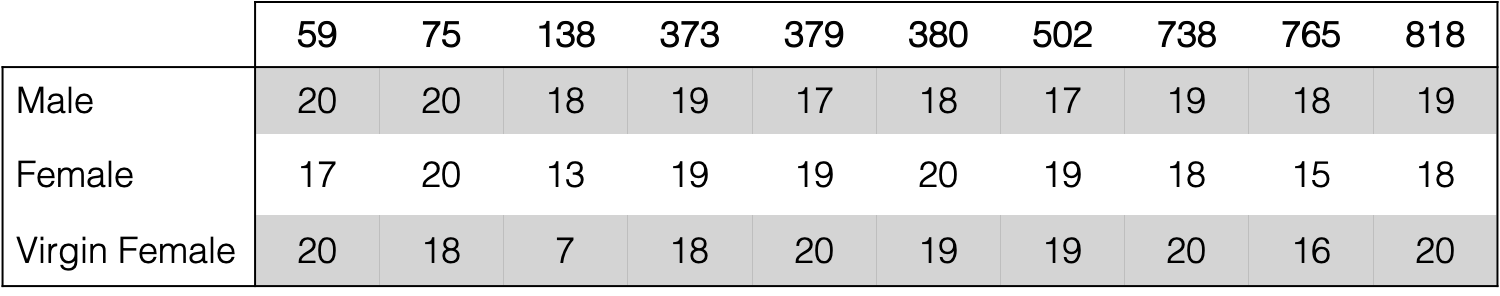


**S1 Table** – The number of flies measured for lifespan and viral load at death for each combination of genetic background and sex/female mating status.
