# Supplementary material for: Dissecting genetic and sex-specific sources of host heterogeneity in pathogen shedding and spread
Source: PLoS Pathog. 2021 Jan 19;17(1):e1009196. doi: 10.1371/journal.ppat.1009196 (PMC7846003; doi:10.1371/journal.ppat.1009196)
Supplement: S3 Table — All interactions are fully-factorial and marked using an asterisk (*). (DOCX) [file ppat.1009196.s004.docx]

| Response Variable | Analysis | Predictors |
| --- | --- | --- |
| Lifespan | GLM | Sex * Genetic Background * VLAD |
|  |  | Mating * Genetic Background * VLAD |
| VLAD | GLM | Sex * Genetic Background |
|  |  | Mating * Genetic Background |
| Qualitative Load | Logistic Regression | Sex * Genetic Background + DPI |
|  |  | Mating * Genetic Background + DPI |
| Quantitative Load | GLM | Sex * Genetic Background + DPI |
|  |  | Mating * Genetic Background + DPI |
| Qualitative Shed | Logistic Regression | Sex * Genetic Background + Quant. Load + DPI |
|  |  | Mating * Genetic Background + Quant. Load + DPI |
| Quantitative Shed | GLM | Sex * Genetic Background + Quant. Load + DPI |
|  |  | Mating * Genetic Background + Quant. Load + DPI |
| Qualitative *V* | Logistic Regression | Sex * Genetic Background |
| Quantitative *V* | GLM | Sex * Genetic Background |

**S3 Table.** Summaries of the logistic regression and GLMs used to analyse the response variables of our experiments. All interactions are fully-factorial and marked using an asterisk (*).
