# Supplementary material for: Dissecting genetic and sex-specific sources of host heterogeneity in pathogen shedding and spread
Source: PLoS Pathog. 2021 Jan 19;17(1):e1009196. doi: 10.1371/journal.ppat.1009196 (PMC7846003; doi:10.1371/journal.ppat.1009196)
Supplement: Attachment — Submitted filename: PLOSPath-Response to Reviewers.docx [file ppat.1009196.s005.docx]

Dear Dr Vale,

Your manuscript has now been reviewed by three well-qualified reviewers. Two reviewers with a primary EEID background appreciate the novelty of the work including the comprehensive nature of the measurements and the utility of the system. They suggest substantial revisions to the manuscript. The third reviewer has an EEID and pathogenesis background. They recommend rejection.

Overall, I think the reviews are fair in their appraisal and accurate in the details of their feedback. Because there is a wide range of opinion on the manuscript, it suggests that rewriting could improve the appeal to the PLOS Pathogens readership.

**I think the comprehensive measurements and calculation of the determinants of inter-individual variation in disease transmission potential are valuable to the field and will enable new work by theoreticians and modelers. Furthermore, establishing such work in a genetics system such as Drosophila could lead to powerful future experiments**. However, the reviewers also brought up methodological questions in terms of how the experiments were designed and executed. A discussion of the experimental logic and how it might affect the results would help. For instance, Reviewer 1 notes that the DCV virus is naturally acquired by feeding but here it is septically introduced. Could that be the cause of the high load, low shedding phenotype?

All three reviewers suggest improvements to the writing that could overcome some deficits. For instance, Reviewer 1 notes that the "lean" writing style could be improved by placing the methods section before the results. Reviewer 2 makes many helpful suggestions as to how the authors could clarify the writing, which would also address some of the concerns of Reviewer 3. Reviewer 3's primary concern was a lack of novelty. More specific clarification of the gaps in our knowledge in the abstract and introduction should be made in order to convey the importance of this work to those outside the field of EEID.

I encourage you to substantially address all of the reviewer concerns and resubmit. In your rebuttal letter, please be sure to carefully address all of the reviewer feedback.

**Response: We have accepted all the reviewers’ suggestions for improvement (see point-by-point rebuttal below).**

**Part I - Summary**


**Reviewer #1:** This study examines the contributions of host sex, mating status, and genetic background to among-host variation in the transmission potential of Drosophila C Virus. The study finds that some of these variables and their interactions account for appreciable variance in transmission-relevant traits like quantitative resistance, viral shedding, and lifespan post infection. The variance in these traits is further used to bootstrap and estimate the distribution of transmission potential among individuals in a population, finding a combination of zero-inflation and a small but potentially epidemiologically important superspreader tail.
**The motivation for this study is excellent – the field desperately needs work that rigorously connects variation in the within-host dynamics (e.g. viral load, qualitative vs quantitative resistance) of infection to between-host dynamics, and this study ambitiously approaches this goal. Thus, I think it is of sufficient novelty and broad interest for the readers of this journal.**
I struggled in my first reading of this manuscript because it is written in an excessively lean style, given that the methods do not appear till the end. Several key “what” and “why” details should be elaborated upon in the results and discussion, to help readers better understand the experimental design and relevance and limitations of the results (specific suggestions below).

**Response:**

**We thank the reviewer for praising the motivation and the novelty of our work. We agree that the field desperately needs work that experimentally dissects individual host heterogeneity and links this to population level disease dynamics.**

**We also agree that the writing of the previous version was not clear and too lean in some parts. In this revised version, we have made several changes we feel have improved the readability of the paper. One major change was to move the methods section to appear before the methods, which should go some way to help the comprehension of the results. In addition, we have re-written much of the results to add greater context (the what and the why) to each result. Finally, we have taken care to address each specific comment raised by the reviewer below.**

**Reviewer #2:** This manuscript aims to explore how three factors of host identity (sex, genetic background, and mating status) interact to produce heterogeneity in pathogen transmission potential. Using a model host-pathogen system (Drosophila and DCV), they found extensive among-individual variation in transmission potential, and a much of this variation is explained by genotype x sex interactions. The experiments are exploratory, but provide interesting empirical data on transmission heterogeneity, and the authors combine these measures with previous data on fly social behavior to estimate V (individual transmission potential) and present these data very well (e.g., Figure 4a). **Overall, the intellectual merit of this paper is twofold: the authors describe and quantify potential factors contributing to pathogen transmission heterogeneity and their interactions, and they also provide a great foundation for future studies using this model host-pathogen system. Drosophila is clearly recognized as a model for physiology, genetics, and pathology, but these types of studies show how powerful this system can be used as a tool to study ecology and evolution of infectious diseases. I think this will make a fantastic contribution to the literature, and will garner broad readership in PLoS Pathogens.**

**Response:**

**We thank the reviewer for their encouraging and complimentary remarks regarding the intellectual merit of our work, and how this work may offer a foundation for future experimental dissection of heterogeneity in disease transmission in this and other systems. Please see below for point-by-point responses to more specific queries.**

**Reviewer #3:** The manuscript by Jc fonathon A. Siva-Jothy and Pedro F. Vale has tested how common sources of variation between individuals (genetic background, sex and mating status) contribute to individual heterogeneity in pathogen transmission potential using Drosophila melanogaster and Drosophila C Virus as a model system.
**The primary weakness of the paper, conceptually and in terms of novelty, is that the effect of host trait on disease transmission has been determined in other host-pathogen system. Indeed, the authors here systemically measure the effect of multiple host traits on disease transmission, which provides new information to fully understand the sources of heterogeneity in pathogen transmission.** Overall the paper is succinct and a thorough study of the effect of host trait variation on pathogen transmission.
However, I have some lingering questions after reading the paper and suggestions for experiments that could improve or substantiate the work presented here.

**Response:**

**Thank you for your constructive thoughts on our work. We are pleased to see the reviewer agrees with reviewers 1 and 2 regarding the novelty of work in measuring multiple host traits that could affect heterogeneity in disease transmission. We were surprised by the reviewer’s (somewhat contradictory) opinion that measuring this variation is somehow conceptually weak and not novel, as these are precisely the aspects of our work that the other reviewers praised for being novel, important, timely and of intellectual merit. We believe this view may in part be due to some of the flaws in our writing in the previous version and we hope our careful revision will change the reviewer’s perspective about our work.**

**Part II – Major Issues: Key Experiments Required for Acceptance**


**Reviewer #1:** I also have three intermediate-to-major concerns about the experimental design that at least deserve substantial discussion or clarification, if additional supporting data is not readily achievable:

**2.01)** I understand that septic infection is convenient in that you can closely control the initial dose (a key source of variation), but this is a fecal-oral virus and you are measuring fecal transmission, correct? Given what is known about the life history of this virus, might you expect different results (e.g. the degree to which different variables and their interactions explain transmission virulence) if infected through the natural transmission mode? Most importantly, would you still predict the same zero-inflated + superspreader distribution? I think the absence of data on this dampens the impact of this paper (but maybe I am missing something about the host-microbe model here).

**Response: While DCV infection has been detected in wild flies (although it is rare), its route of transmission is not entirely clear. It is horizontally transmitted and the route is very likely to be fecal-oral because it shows tissue tropism for the crop and midgut epithelia and is often found to be a lab contaminant in many stock lines.**

**We did initially consider the possibility of doing these experiments using oral infection. We have previously investigated the fitness consequences of oral infection in this system (see Gupta, et al. 2017 *Journal of Evolutionary Biology* 30: 1325–35.) so this would in principle have been possible. One considerable drawback is that we have found that extremely high concentrations of DCV are required to establish a lethal infection when administered orally, on the order of 10^10^ viral copies per ml (and this is confirmed by other groups trying the same infection route, see Ferreira et al 2014 Plos Pathogens** *10*(12), e1004507**). For an experiment of the scale we present here, this would require culturing enormous quantities of virus, which was not feasible for us.**

**Another - more important - shortcoming of this methodology is that it is difficult to control the amount of virus ingested by flies. Were this experiment to be repeated using oral, rather than systemic infection, we would therefore expect a number of differences in our results, however it would be difficult to discern the extent to which these differences were caused by variation in infectious dose ingested by flies or the difference in transmission route.**

**In defence of the systemic route of infection we chose, it is repeatedly observed that even using systemic infection (thoracic or abdominal pricking/injection) , the main focus of viral infection is the crop and midgut, indicating that DCV shows tissue tropism for the gut . The fact that we are then also able to detect DCV in fecal shedding lends support to the idea that it is still possible to measure viral shedding following a systemic infection. We believe it would still be likely to observe a zero-inflated distribution, simply because this overdispersed, zero-inflated distribution is repeatedly observed in pathogen shedding across a wide range os host species and pathogen types see f.e.,**

1. **Wilson et al (2002) Heterogeneities in macroparasite infections: patterns and processes. The Ecology of Wildlife Diseases. Oxford University Press. 197 p**
2. **Woolhouse, Mark EJ, et al. "Heterogeneities in the transmission of infectious agents: implications for the design of control programs." *Proceedings of the National Academy of Sciences* 94.1 (1997): 338-342**
3. **Lloyd-Smith, et al “Superspreading and the Effect of Individual Variation on Disease Emergence.” *Nature* 438, no. 7066**

**However, we agree that this route of infection is likely to have impacted the zero inflated correlation between viral load and viral shedding (Figure S1) for two reasons. First we are necessarily comparing the viral load in the whole fly to only the fraction of virus which makes it to the gut and is then excreted. Second, there is likely to be a time lag between growth in the hemocoel, migration to the gut and finally externally shed virus and therefore the lack of strong correlation between the two measures is likely exaggerated by the route of infection. We have added an entire section to the discussion of potential caveats in the discussion (L501-519)**

**2.02)** The intra-fly correlation between lifespan and viral shedding is not clear, because very few flies (even those with measurable viral loads) die before 10 days, but shedding is only measured in the first three days. Viral Load at Death is measured when the flies die, but there is no significant correlation between viral load and viral shedding (supp. figs). So, how does variation in lifespan really speak to the transmission period in this model? If this was carefully considered, it is not clear from the text.

**Response: We would like to thank the reviewer for raising this issue as it is also clearly the source of a number of minor comments as well. Indeed, this comment highlights that our description of what was measured and when it was measured was not clear, because we did not present any correlation between lifespan and viral shedding. We believe the reviewer is referring to figure 1C, which shows the correlation between lifespan and the viral load at death (VLAD). This was the internal viral load of the fly recorded on the day it died, not the number of shed viruses. Shedding shown in the following figures was measured within the first 3 days, and this period was chosen because we have repeatedly found that in systemically infected flies DCV titers increase and then stabilise following 3 days, so we took this as likely to be the period when most of the viral shedding might occur.**

**Most of the concerns raised in this comment can be addressed by a clearer outline of our experimental design and the motivations behind it. We have now added a more detailed description of both the experimental design the aims of these experiments to the introduction section of this paper. These descriptions outline that in one experiment, infected lifespan and viral load at death were measured in the same flies, and in a second experiment, we measured viral shedding and viral load in the first 3 days. We only test the correlation between traits measured in the same individual (infected lifespan with viral load at death, and viral load with virus shedding). To provide further detail on our experimental design, and in accordance with a number of other comments made by reviewers, we have also moved the methods section of this paper so that it now follows the introduction.**

**2.03)** Is the estimated heterogeneity in transmission potential really reflective of a natural population? Fig. 4 distribution shows a lot of variance, but it is worth noting that the data are from the 5 most resistant and susceptible Dmel lines, meaning that the variance has been artificially maximized. The distribution in a natural heterogeneous population is likely much lower.

**Response: Our aim was to use a panel of fly lines with a range of susceptibilities present within the DGRP panel. We would disagree that the variance is artificially maximised, as while we picked lines that were relatively resistant / susceptible, the variation we see in DCV susceptibility (Fig 1a) is relatively continuous and is representative of the population’s range in susceptibility. If the lines we picked are present in the wild, we do not see how the** distribution in a natural heterogeneous population is likely much lower**. Going beyond this point, it is generally difficult to generalise from 10 lines to a natural population. Our aim was not necessarily to replicate the levels of variation present in the original DGRP population, but simply to study how traits involved in pathogen transmission will vary in 10 distinct genetic backgrounds.**

**Reviewer #2:**

**2.04)** I think the term “superspreader” should be de-emphasized in the introduction (and removed from the keywords). Although superspreader events are an extreme form of transmission heterogeneity, this paper does not provide any direct empirical evidence for how superspreader events can be generated by differences in host traits. I think that future studies could certainly use this system to do so, and this paper certainly sets the groundwork for those experiments.

**Response: The reviewer circles back to this point in comment 3.30, where they then suggest expanding on the superspreader concept more in the discussion section as a solution to this comment. Specifically, we now emphasise the long-tail distribution of *V* and link this more overtly to extreme individuals. We also draw tentative parallels between the extreme individuals in our experimental treatment groups and superspreaders in more natural populations.**

**2.05)** The abstract might be more meaningful if the authors included some quantitative descriptors of their results, perhaps some of the variance explained by sex, genotype, mating status, or the effect size/magnitude of change found between sexes or the most different genotypes. Saying that one genetic background had X% higher viral shedding, for example, than another is more informative than just saying there are vast differences between genotypes.

**Response: Thank you for this suggestion. We agree that our abstract needs to place more emphasis on these differences. We have now added a more detailed explanation of our findings in the abstract and accompanied descriptions of our findings with examples of notable levels of variance being explained by the interaction between genetic background and sex. For our experiments, these focus on traits where genetic background alone, or the interaction between genetic background and sex, explain a lot of variance, and for our estimations of *V*, we focus on quantitative variation.**

**2.06)** I suggest that the authors be more forthright in the introduction that this is exploratory research aimed at discovering the degree of heterogeneity in pathogen transmission potential, rather than testing hypotheses about specific genotypes, sexes, or their combinations. Given the huge amount of work involved in these experiments, this isn’t a criticism by any means, this type of exploratory work is very important, but the results will be put in a clearer context if the reader knows these aims by the end of the introduction.

**Response: Thank you for this suggestion. In order to emphasise the exploratory nature of this work we have now added a brief description of our intent to the introduction.**

**2.07)** It would be helpful if the authors expounded a bit on the difference between qualitative and quantitative values of shedding. I understand how you measured these things, but what they mean in the context of biology is still unclear.

**Response: A number of other reviewer comments voiced a similar concern that relate to this point, and we have now added a series of descriptions in the results section (where we first introduce our distinctions between qualitative and quantitative variation).**

**2.08)** Since one of the goals of the paper is to show how this system is a valuable model for studying transmission heterogeneity, I would have liked to see some more information about the natural history of the interaction between Drosophila and DCV, if there is anything known about prevalence in the wild, evidence for epizootics, etc.

**This is a very good point, thank you. We have now included a section on lines 134-140.**

**Reviewer #3:**

**2.09)** It has been reported in many host-virus systems that genetic and sex-specific variation can affect individual heterogeneity in different components of disease transmission, which is interpreted again in this study, my major concern of this study is the lack of novelty.

**Response: We appreciate the reviewer’s concern. The key novelty of this work is that we quantify the effects of genotype and sex on several key traits that determine pathogen transmission, and further we then combine these into a single composite measure of transmission potential V. Indeed, our aim was break down transmission into its component parts, quantify the level of variation in each part with the aim of showing that this is viable model system to investigate heterogeneity in disease spread. This novelty was also highlighted by reviewers 1 and 2, and it something that recent literature has highlighted as a desirable direction in the study of disease transmission (**VanderWaal, Kimberly L., and Vanessa O. Ezenwa. "Heterogeneity in pathogen transmission: mechanisms and methodology." *Functional Ecology* 30.10 (2016): 1606-1622.)

**2.10)** In the main text, only mated females were measured, there is no explanation for not characterizing mated males.

**Response: We included three groups of flies: Virgin females; mated females; and mated males. Sex differences were made between mated males and females, while the effect of mating was investigated by comparing virgin and mated females. We investigated the effect of mating on females only because the effects of mating on immunity are well documented in female Drosophila. To ensure this methodology is clearer the methods section now follows our introduction and contains a more detailed description of this methodology.**

**2.11)** Figure 2a: do you have any ideas that the proportion of flies with non-zero DCV load differed between virgin and mated females from particular genetic background? It should be somehow discussed.

**Response: This is a great point, thank you. The analysis of proportion of flies with detectable DCV loads (the qualitative DCV load) is shown in Table 3. The question of whether the difference in this proportion due to mating status varied with genetic background is therefore given by the interaction between mating x genetic background in that model, which is significant (Table 3). Looking at figure 2a, these effects appear to driven by a few extreme differences between mated and virgin females (e.g. line 818 at day 3, or line 765 at day 2). While these effects appear large they are far from consistent and often transient, only occurring on one day for example. So we thought it best not to make a huge deal out of this effect without understanding the reason for this transient nature.**

**Part III – Minor Issues: Editorial and Data Presentation Modifications**


**Reviewer #1: Other Comments
3.01)** Lines 105-107: More rigorous examples where covariance among traits (other than just the anecdotal typhoid Mary and SARS) would help to underscore the existence of a gap in knowledge here. Also, what is “coupled heterogeneities?” That sounds like a term that I would use as filler if I didn’t know quite what I wanted to say. I think it needs clarification.

**Response: Thank you for this suggestion. We have now added an additional explanation of what we mean by coupled heterogeneities - the need to quantify heterogeneity not only in single traits but how multiple traits co-vary to influence transmission dynamics. Far from being filler, it is a concept developed in some depth see references below and now cited in the main text. We now add a better explanation with specific examples of other empirical studies that measure such co-variances (112-121).**

**(see: Vazquez-Prokopec, et al (2016). Coupled heterogeneities and their impact on parasite transmission and control. Trends in parasitology, 32(5), 356-367.**

**And**

**White, L.A., Forester, J. D. and Craft, M. E. (2018). The role of host heterogeneity in determining epidemic outcomes: Covariation between the physiological and behavioral components of transmission. Oikos, 127(4), 538-552. doi:** [**1111/oik.04527**](http://onlinelibrary.wiley.com/doi/10.1111/oik.04527/full)

**3.02)** Lines 107-108: I would change "fully" and "essential" in this sentence; measuring multiple traits may still not allow you to fully understand, and conversely a full understanding may not require the measurement of multiple traits (perhaps one trait explains an outsized amount of variance)

**Response: Thank you, we have now changed essential to important.**

**3.03)** Lines 117-120: I think it is essential here that you note which measurements come from the same individual flies (e.g. allow you to get to the intra-fly covariance) and which are relying on averages among fly groups. For better or for worse, the impact (and the most important limitations and reservations, to my mind) come from the extent to which these measurements are coupled across time and within flies.

**Response: Excellent suggestion, which helped add clarity in the description of our experiments. To help the reader understand which traits were measured in the same flies, and provide an insight into intra-fly covariation, we have rewritten this section at the end of the introduction L141 onwards.**

**3.04)** Intro: I think it would be helpful somewhere in here to introduce the concept of quantitative vs. qualitative infection outcomes (including biological examples of systems where this is an important consideration), since it becomes so central to the analysis and interpretation of the results.
Results: Overall, the results needs more of an introduction to the general experimental design. How is oral infection carried out? How many genotypes did you infect? How long does it take flies to die, in general? When did you measure viral load? If the methods come at the end of the document, these details need to be here, to provide necessary narration. Otherwise just put the methods before the results; surely PLoS has some leeway there.

**Response: We have made a number of changes to the paper to outline the differences between, and relevance of, qualitative and quantitative variation in viral load and virus shedding, respectively. These descriptions and details have been added to the results section to accompany descriptions of the statistical tests. In addition, we have also moved the methods section to follow the introduction to outline and place more emphasis on our experimental design, and rewritten much of the results section to provide better context and narration, which was admittedly sorely needed.**

**3.05)** Lines 126-127: Some summary statistics would be helpful here. On average, how much did infection reduce lifespan?
Fig 1A: Kind of hard to eyeball general trends across columns here, other than they aren't precisely rank ordered

**Response: Thank you for this suggestion, we agree that summary statistics would help the reader understand the key take-home messages of our results. To keep the focus on variation in host response to infection, we have now included in the results section, a comparison between the highest, average and lowest changes between males and females in lifespan.**

**3.06)** Line 133: Interesting. Is this because later-dying flies started clearing infection and then died, or do you think that cumulative load, rather than peak load, explains mortality?

**Response: Our interpretation of these data is that the flies that died sooner represent individuals less able to limit viral growth, and as a result experience higher mortality. However, without a clearer picture of viral growth over the course of infection, particularly one in which viral load is measured longitudinally in single flies, it is hard to conclusively distinguish between those two scenarios.**

**3.07)** Line 136: Were they not properly infected/colonized or did they clear infection? This section would benefit from a sentence or two introducing the experiment in question, since the methods are at the end of the document

**Response: We agree with the reviewer that further explanation would benefit the reader’s understanding of these results. We have now added a brief description of our experimental infection protocol. Given that many flies with undetectable load showed detectable and often substantial amounts of DCV in their shedding vial (Figure S1), it seems unlikely that they weren’t properly inoculated (which is unlikely when pricking a virus covered needle directly into the thorax). Instead it seems more likely that these flies were clearing and/or expelling DCV into the vial.**

**3.08)** Line 140: Should this be labeled as “detectable” instead of ‘non-zero’ on the Fig. 2a axis?

**Response: We agree that changing non-zero to detectable is clearer to the reader and have now amended throughout the manuscript.**

**3.09)** Line 141: These are collected in first three days after infection. Do flies start to die before this? Do they start to die well after this? What does DCV titres at this stage really tell you?

**Response: Our primary motivation for measuring viral titres in flies during the first three days of infection was previous work showed viral titres increase and peak by day three.**

**3.10)** Line 141; Fig. 2a: Not clear from just looking at the figure that those bars are time points. Why not represent the data in the same way that you represent the quantitative data, or show more of a binomial style of plot with actual data points?

**Response: Thank you for this suggestion. We plotted this data as bars to clearly distinguish between qualitative variation from quantitative, and as these results are proportion data, bars would be the most appropriate way to present this information. However, we have now changed the x-axis labels to days post infection and added a description into the figure legend that the colour of the graph’s bars denotes to sex. This layout still allows sex/mating to be easily discerned but also clearly indicates time on the x axis.**

**3.11)** Line 149-150: Why are these pairwise? Is this better than a quadratic model?

**Response: Fitting a quadratic model to 3 timepoints isn’t really great, or certainly not better than treating time as a categorical factor. Also, the viral load and shedding of days 1, 2 and 3 were measured in different flies (because sampling of internal viral loads is necessarily destructive) so we felt it more appropriate to analyse daily variation in these traits as categorical, rather than continuous variables.**

**3.12)** Line 156: How is shedding measured? Give us a crumb of a method here.
**Response: We have now added a sentence at the start of this paragraph that briefly summarises our collection method.**

**3.13)** Line 176: did you use individual distribution over time curves for this?

**Response: We’re unsure what the reviewer means with “individual distribution over time curves What we did, which we have made clearer by altering the first few sentences of this paragraph and prefacing our results section with the methods section which provides a more detailed explanation of how we designed this framework (L373) was :**

***To generate an estimate of an individual’s transmission potential, V [2,13], we combined the single measures of lifespan and virus shedding (1, 2 or 3 DPI) described above with previously published data on social aggregation in males and females of the same genetic backgrounds [27]. By simulating theoretical “individuals” where each trait value was sampled from these experimentally derived distributions of lifespan, shedding and asocial aggregation, we obtained a distribution of the variation in individual transmission potential V (Fig. 4A).***

**3.14)** Line 180: Fig. 4a distribution is interesting. What does R0 mean for a proportion? Not quite sure I get the x axis there.

**Response: In response to another reviewer comment regarding figure 4a we have now removed the R0 notation. However, we would like to thank the reviewer for raising this issue as it has drawn attention to our need to further explain the relevance of V/Vmean. We have now included a clearer explanation for making our measure of transmission potential relative to the population average in the methods section, and in the legend of figure 4a.**

**3.15)** Line 180, and Line 293: These paragraphs need some substantial revising here and there for grammar

**Response: We have revised the writing in the entire manuscript substantially.**

**3.16)** Line 208-9: Do those that fail to shed also survive? If not, could there be shedding post mortem?

**Response: This is very good question. Given we only measured how much DCV flies shed once (so that we could sample their DCV load on the same day, and this sampling is destructive), we do not know how shedding relates to lifespan following infection. With respect to shedding post mortem, infected fly carcasses have been shown to be a potential source of transmission to cannibalistic larvae eating dead adults however the transmission risk these carcasses bear to other adults is unclear. But is seems possible there would be some shedding post mortem.**

**See: Siva-Jothy, J. A., Monteith, K. M., & Vale, P. F. (2018). Navigating infection risk during oviposition and cannibalistic foraging in a holometabolous insect. *Behavioral Ecology*, *29*(6), 1426-1435.**

**3.17)** Lines 235-61: This paragraph is long and starts to get a little list-like

**Response: We agree with the reviewer that we can improve the writing here. We have now refocussed this section and rewritten this paragraph as two in order to detail variation in transmission and the genetic basis of shedding and how they have been studied in the literature.**

**3.18)** Line 267: Would it be worth it to show male vs female V/Vmean distributions, perhaps as overlapping in Fig. 4A?

**Response: Thank for this recommendation, we have now edited this figure panel to display the distributions of the male and female populations separately (although as the reviewer will notice, there is considerable overlap between sexes).**

**3.19)** Lines 281-288: I appreciate the connection to other systems, but the testosterone discussion roams pretty far from being relevant for your results. AND **3.20)** Lines 289-90: This example just hangs there. Are you trying to make a point about vertebrate vs. invertebrate sex bias?

**Response: It was our intention to briefly outline the other findings in the literature that have characterised sex-specific variation in behavioural and physiological traits. We have now reworded this section to read as a brief summary of findings across a range of systems as opposed to a direct comparison between them.**

**3.21)** Lines 312-313: Do we know what that dose response looks like? Is it threshold-like? Could R0 actually be lower than estimated?

**Response: The short answer is we don’t know, especially since this would require a series of oral infections at rather low doses, which Is technically not straightforward (we have tried and repeatability is an issue when working close to DCV qPCR detection limits). Characterising how host shedding is affected by infectious dose would be a really interesting avenue of research, offering vital insight into the significance of the amount of virus shed with respect to transmission to susceptible individuals. Together, the influence of infectious dose and the variation in shedding may affect transmission of DCV in the wild, however understanding the extent of this influence lies well beyond the scope of this paper. Instead the work presented intends to characterise how genetic and sex-specific variation in multiple traits could affect transmission potential when present in the same individual.**

**3.22)** Lines 318, and surrounding: I think there needs to be a deeper and more thoughtful discussion of how sampling limitations and within-group variation reflect on covariance among variables

**Response: This section (now L500 and onward) has now been substantially revised to discuss the caveats of the experimental design.**

**3.23)** Lines 340-1: Which 5 and 5 genotypes? Did your results recapitulate this?

**Response: Thank you for highlighting this oversight, we have now added a brief description of the resistant and susceptible DGRP lines used in the methods section and made mention of our findings corroborating those of Magwire *et al*. 2012 in the results section.**

**3.24)** Line 362 and Line 373: This is not a huge sample size, given the clear variation in clearance rates and zero-inflation. Probably sums up to a good sample size for single variables, but the interaction effects might be underpowered (e.g. some genotype x trait groups have an N=1). Also, was the experiment repeated multiple times? Where do the error bars come from?

**Response: The points on the graph show means and standard errors of the replicates within each of treatment groups. We appreciate that in some of these treatment the sample sizes are not as large as would be desired, especially when analyzing 3 way interactions. We are unfortunately susceptible to the same limitations of resources (particularly time, money and manpower), that would otherwise allow us to make every experiment 10 times bigger. The criticism though is still very valid, which is why we do not try to make big inferences on unique genotype x time x sex effects, but rather focus on the larger and statistically more robust effects of genetic background and sex.**

**3.25)** Line 370: Did you have a group of flies that were measured for only load without the shedding assay first, to see if it was stressful and/or made a difference for the load results?

**Response: Thank you for raising this interesting point. We tested if housing flies in Eppendorf tubes was stressful only by testing if they survived for a few days. While it is possible that this environment may have impacted their viral growth and shedding, it would have affected all flies and they all experienced these conditions for the same amount of time across both sexes and genetic backgrounds.**

**3.26)** Fig S1: I think Fig. S1 labels are backwards - shouldn't high load but low VLAD individuals be supersponges?

**Response: Thank you for spotting this, it has now been corrected. Again, this is not VLAD, but shedding, but the correction remains valid.**

**Reviewer #2**

**3.27)** Lines 14-15: The abstract sets up the paper as if you will measure the se underlying effects, but rather you manipulate host genotype/sex/mating status and find widespread effects on transmission potential. However, the mechanisms remain unknown. An extra sentence in the abstract to explain that factors of host identity like genetic background and sex can influence these underlying mechanisms that then influence transmission potential.

**Response: Thanks for this helpful suggestion. We have now added a more detailed explanation to the abstract, to help the reader understand the focus of this paper being to characterize sources of host variation that may impact upon transmission potential.**

**3.28)** Line 88: Since immune traits are a form of physiological traits, I would replace that with “social factors” or something to point out that transmission is influenced by the interaction between infected hosts and susceptible hosts. A relatively recent paper showed that social context can be a predictive factor of disease risk in Drosophila social aggregations:
Keiser, C.N., Rudolf, V.H., Sartain, E., Every, E.R. and Saltz, J.B. 2018. Social context alters host behavior and infection risk. Behavioral ecology, 29(4), pp.869-875.

**Response: Perhaps this is unclear in our original sentence, but our reference to behavioural traits that affect transmission are predominated by social interactions, such as sociality or behavioural changes following infection. To clarify this link to the reader we have amended this sentence to include a brief description of how host traits affect the core components of disease transmission.**

**3.29)** Line 141: DPI should be defined before its first usage here, as well as what you mean by “DPI effect”.
**Response: We agree that our reference to a DPI effect is redundant as we have already stated there is a significant effect of time on the presence or absence of viral load, we have now deleted this term.**

**3.30)** Line 182: Ah, here we see some evidence for potential superspreaders. “Long-tail” distributions like this are common in many diseases, and the individuals are the far right end may represent superspreaders (as described in Lloyd-Smith’s Nature paper from 2005). If the authors prefer to keep their references to superspreaders in the introduction (see my comment #1), then this should be highlighted in the discussion more explicitly.

**Response: We would very much like to maintain our emphasis on superspreaders as we feel our work on characterising heterogeneity is directly linked to this field. To that end we have placed more emphasis on the ideas presented in Lloyd-smith, particularly in the discussion where we discuss the potential for females of RAL-380 to act as superspreaders.**

**3.31)** Line 185: Statements like this, and throughout the results section, would be much more powerful when they include some effect size – what was the sex-difference in proportion here?

**Response: A similar issue was raised by reviewer comment 2.05 which requested more detail about the differences between our treatment groups. We now place more emphasis on the percentage of variance explained by specific variables in the abstract and throughout the paper.**

**3.32)** Lines 242-261: This seems like a laundry list of studies that showed genotypic effects on pathogen shedding/transmission – it might read a little easier if they were distilled into main take-away messages, and how they help explain the effects discovered in your experiments.

**Response: Thank you for making this suggestion. This section has now been rewritten as two paragraphs to place more emphasis on shedding and transmission separately.**

**3.33)** Line 267: How is this statement different from the opening sentence of this paragraph?

**Response: Thank you for pointing out this repetition, this sentence has now been rewritten to emphasise that our findings align with the differences between male and female disease transmission seen elsewhere in the literature.**

**3.34)** Line 279: There are also studies on Drosophila sex-differences in pathogen avoidance, and how that is related to susceptibility (e.g., Keiser et al 2019, Sex differences in disease avoidance behavior vary across modes of pathogen exposure; Ethology; and references therein). How avoidance behavior related to transmission potential might be an interesting addition to this paragraph.

**Response: Thank you for making this reference suggestion, we agree that Keiser et al. 2019 is great fit to this section. This paragraph has been quite extensively rewritten in response to another reviewer’s comments (#3.19 and #3.20), and it is now a broader round-up of sex-specific variation in traits involved in transmission. Part of this summary now makes reference to the Keiser paper’s findings of sexual dimorphism in avoidance behaviour and how this is seemingly linked to variation in susceptibility.**

**Reviewer #3**

**3.35)**The paper has used extensive long sentences without the proper commas resulting in difficulties to follow. It can be simplified by reducing the use of complex sentence structures.

**Response: We appreciate that our writing could have been improved and in this revised version we have taken efforts to improve the grammar and narrative.**

**3.36)** Figure 1a&b: statistics should be noted for the two figures either in main text or in the legend.
**3.37)** Figure 2a&b: statistics should be note, and mark for significance should be denoted.
**Response: We carefully considered the best way to present these data. Given the multiple treatments it is tricky to present significance within the figures as there are multiple comparisons of interest. Including p-values in the text is one option, but makes the text very dense and clunky and breaks up the prose. Instead we felt the most complete way to provide all statistical information in tables indicating the main effects, p-values and (more important even than p-values) the % variance explained by each effect in the model.**

**3.38)** Carefuly check the writing, such as in Line 161, please carefully check ‘while these were effects were significant’.

**Response: We would like to thank the reviewer for drawing our attention to these errors. We have proof-read the paper again to correct these mistakes.**

**3.38)** The authors should carefully check the format of reference and keep it consistent, for example, for some journal name, abbreviations were used. The paper also excessively cites publications; less than half of the citations are likely necessary.

**Thank you for taking the time to point out these annoying inconsistencies in our references, driven mainly by the vagaries of reference management software. Regarding the excessive nature of our referencing, we have been careful to cite adequately and to cite the appropriate literature without excess. The fact that we are dealing with behavioural, physiological traits, sex differences, mating effects all within the context of disease ecology and pathogen transmission means that we are bound to cover several areas of research that deserve mention. In fact, many of the reviewer comments above have requested even further examples and additional references. It is fortunate that PLos do not impose draconian limits on such things, so we are happy to use this to cite appropriately.**

**3.39)** Line 314, the format of the two dashes is different.

**Response: Thank you for bringing this to our attention. We have now matched these dashes.**
